# Implementing nutrition guidelines for older people in residential care homes: a qualitative study using Normalization Process Theory

**DOI:** 10.1186/1748-5908-7-106

**Published:** 2012-10-30

**Authors:** Claire Bamford, Ben Heaven, Carl May, Paula Moynihan

**Affiliations:** 1 Institute of Health and Society, Baddiley Clark Building, Newcastle University, Richardson Road, Newcastle, NE2 4AX, UK; 2 Faculty of Health Sciences, University of Southampton, Southampton, UK; 3 Institute for Ageing and Health/Centre for Oral Health Research, School of Dental Sciences, Newcastle University, Newcastle, UK

**Keywords:** Normalization process theory, Nutrition policy, Guideline, Long-term care, Older people, Qualitative research

## Abstract

**Background:**

Optimizing the dietary intake of older people can prevent nutritional deficiencies and diet-related diseases, thereby improving quality of life. However, there is evidence that the nutritional intake of older people living in care homes is suboptimal, with high levels of saturated fat, salt, and added sugars. The UK Food Standards Agency therefore developed nutrient- and food-based guidance for residential care homes. The acceptability of these guidelines and their feasibility in practice is unknown. This study used the Normalization Process Theory (NPT) to understand the barriers and facilitators to implementing the guidelines and inform future implementation.

**Methods:**

We conducted a process evaluation in five care homes in the north of England using qualitative methods (observation and interviews) to explore the views of managers, care staff, catering staff, and domestic staff. Data were analyzed thematically and discussed in data workshops; emerging themes were then mapped to the constructs of NPT.

**Results:**

Many staff perceived the guidelines as unnecessarily restrictive and irrelevant to older people. In terms of NPT, the guidelines simply did not make sense (*coherence*), and as a result, relatively few staff invested in the guidelines (*cognitive participation*). Even where staff supported the guidelines, implementation was hampered by a lack of nutritional knowledge and institutional support (*collective action*). Finally, the absence of observable benefits to clients confirmed the negative preconceptions of many staff, with limited evidence of reappraisal following implementation (*reflexive monitoring*).

**Conclusions:**

The successful implementation of the nutrition guidelines requires that the fundamental issues relating to their perceived value and fit with other priorities and goals be addressed. Specialist support is needed to equip staff with the technical knowledge and skills required for menu analysis and development and to devise ways of evaluating the outcomes of modified menus. NPT proved useful in conceptualizing barriers to implementation; robust links with behavior-change theories would further increase the practical utility of NPT.

## Background

Despite receiving 24-hour care, older people living in care homes (long-term care facilities, including nursing and residential homes) remain vulnerable to malnutrition. International estimates of the prevalence of malnutrition vary according to the level of care and method of assessment but range from 14%–65% in nursing homes (see [1-3]). Longitudinal data on residential care homes (where people live permanently with 24-hour housekeeping and personal, but not nursing, care) suggest that malnutrition is increasing—from 18.5% in 2004 to 26.2% in 2007 in the Netherlands [4] and from 22% in 2007 to 41% in 2011 in the United Kingdom [5,6]. Malnutrition has significant negative impacts on the physical and emotional well-being of older people, including increased mortality and vulnerability to infections, clinical complications, depression, anxiety, and decreased quality of life [7,8]. The healthcare costs of treating adults with malnutrition have been estimated to be twice that of managing non-malnourished patients [9]. When social care costs are also included, malnutrition is estimated to cost £13 billion annually in the United Kingdom [10]. These costs are disproportionately incurred by clients in care homes; while only 5% of older people in the United Kingdom live in care homes [11], one-third of the healthcare costs of malnutrition in the United Kingdom is for this client group [12].

Interventions to improve nutritional status in long-term care facilities have included education programs [13,14], provision of snacks and/or oral supplements [15-20], and improvement of mealtime ambience and assistance [21-23]. Relatively little attention, however, has been paid to the food provided, although nutritional analysis has shown that such food is typically high in saturated fat, salt, and added sugars, with low levels of starchy carbohydrate and fiber [24-27]. Additional concerns have been raised over the levels of minerals and vitamins provided and whether residents eat enough to meet their energy requirements [24]. Maintaining good nutrition in older people can be challenging since their vitamin and mineral requirements remain stable or even increase, but their energy requirements and appetite decrease. A nutrient-dense diet, adequate in energy, is required; however, few practical guidelines on achieving this type of diet in care homes are available [7,26]. The UK Food Standards Agency (FSA) therefore devised nutrient- and food-based guidance (hereafter referred to as “nutrition guidelines”) specifically addressing the needs of older people (aged 75+) in residential care [28]. Reviews of guideline implementation in healthcare settings have identified a range of factors influencing implementation (*e*.*g*., [29,30]), however, few have considered nutrition guidelines. Factors influencing implementation of nutrition guidelines in intensive care units include guideline characteristics, the implementation process, institutional factors, provider characteristics and attitude, and the clinical condition of the patient [31]. Barriers to the implementation of nutrition guidelines in schools include lack of funding, lack of leadership, student preferences for “unhealthy” food, and perceived goal conflict [32-34]. These studies suggest that factors influencing the implementation of nutrition guidelines may be context dependent and the relevance of existing research to residential care homes is unclear.

In this paper, we report a process evaluation of an uncontrolled before and after study of the impact of nutrition guidelines on the nutrient profile of the food provided and consumed by clients in residential care homes [35]. The nutrition guidelines can be considered to be a complex intervention since implementation involves a range of behaviors and staff with different roles [36]. Process evaluation facilitates the understanding of why and how interventions are and are not successful [37]. Our aim was to understand facilitators and barriers to implementation of the nutrition guidelines and to use this information to optimize the implementation process. The potential value of theory to inform implementation has been emphasized [38,39]; however, to date the majority of studies using theory have relied on rational actor explanatory models [40-42] and greater use of models addressing organizational issues and interaction has been suggested [43]. This study used a novel explanatory framework—Normalization Process Theory (NPT) [44]—which focuses on the social processes and work that people do, individually and collectively, to make an intervention work [44,45]. Four distinct types of practical work are included in NPT: coherence—making sense of the intervention; cognitive participation—investing in the intervention; collective action—the practical work of implementation; and reflexive monitoring—modifying and embedding the intervention [46]. Previous work on NPT has focused on healthcare settings [45,47-49]; we know almost nothing about the implementation of complex interventions in social care settings [43,50], and this paper is the first to our knowledge to have explored this topic using a robust theoretical framework.

## Methods

### Study design

We used qualitative methods (semistructured interviews, informal discussions, and nonparticipant observation) to explore facilitators and barriers to the use of nutrition guidelines in residential care homes. Homes were recruited sequentially, allowing findings from initial homes to inform implementation in subsequent homes. In each home, nutritional data were collected on the menu in use and client intake at baseline. This was followed by a period of menu development during which the study dietitian worked with the cooks to modify menus and recipes to be compliant with the nutrition guidelines. Follow-up nutritional data on the menu in use and client intake were collected at 1, 5, and 12 months following implementation of the modified menus [35]. Data for the process evaluation were collected at baseline, during menu development, and one month after implementation of the modified menus in all homes. Five-month follow-up data were collected in four homes. This enabled us to explore perceived and actual barriers to implementation. The impacts of the nutrition guidelines on the nutrient profile of the food provided and consumed by clients are reported elsewhere [35], but key results are highlighted in Table 1, showing the impacts of implementation in participating homes. Formal evaluation of the impact of the implementation strategies was outside the scope of the study; our intention was to iteratively develop an implementation process addressing key barriers.

**Table 1:** Overview of the context, process of implementation, and outcomes in participating homes

	**Home 1**	**Home 2**	**Home 3**	**Home 4**	**Home 5**
**Context**	Deprived ex-mining community	Isolated rural community	Strong management support	Unsettled staffing with unfilled posts	Study coincided with consultation regarding closure of the home
	Pride in existing menus	Strong resistance to external guidelines	Located on city outskirts	Rural setting	Health-conscious staff
	Compliant staff	Pride in existing menus	Keen to update menus	Changes coincided with appointment of new manager and new cook	Less emphasis on home cooking`
		Empowered staff	Manager and head cook have experience of Slimming World^1^		
**Institutional support**	Manager required cooks to adhere to new menus but provided little support for cooks in dealing with negative feedback	Manager delegated all responsibility to cooks and study team	Manager supportive of guidelines and required cooks to adhere to new menus	New manager keen to change menu structure	Manager instrumental in identifying key members of care staff to contribute to the process of menu development but otherwise had little hands-on involvement
				New manager undermined implementation of guidelines by making changes based on her own preferences and ideas	
**Approach to introducing modified menus**	Menus devised by study dietitian; emphasis on *implementing* the nutrition guidelines	Some attempt to engage cooks in process of menu development but insufficient time to achieve ownership	Emphasis on *working towards* the nutrition guidelines	Emphasis on *working towards* the nutrition guidelines	Emphasis on *working towards* the nutrition guidelines
		Menus largely devised by study dietitian	Majority of menu development carried out by cooks	Majority of menu development carried out by new cook	Care staff involved in process of menu development
		Emphasis on *implementing* the nutrition guidelines	Variable levels of involvement of cooks		Cooks happy to let study dietitian take the lead
					Emphasis on changing recipes rather than dishes
**Outcomes**	Cooks working to rule and abdicating responsibility for menus to the study team	Cooks refused to implement modified menus	Adherence to modified menus varied between cooks	Old cooks still providing cover tended not to stick to modified menus	Changes largely unnoticed by clients
	Modified menus perceived as too extreme and restrictive	Limited changes made	Emphasis on *sticking rigidly* to modified menus	Some dissatisfaction amongst clients	Some variability between cooks
	Reported client dissatisfaction	Some cooks implemented changes in ways intended to fail	Little room for cooks to exercise judgement	Cooks modified menus in light of client feedback	Care staff more engaged and supportive of changes
	Problems with loose bowels due to rapid increase in fiber	Client resistance to changes in the menu	Tendency to revert to old dishes where new dishes unpopular rather than modifying recipes	New manager changed menus while head cook on holiday in line with her own preferences and views	
	Cooks waiting for study team to leave before devising new hybrid menus		Reduction in number of client falls reported by manager		

^1^Slimming World is a UK slimming organisation.

Ethical approval for the study was obtained from Newcastle and North Tyneside Committee (2) of the National Research Ethics Service (07/H0907/170). All participants gave written informed consent.

### Setting

Five public sector residential care homes participated in the study. The homes were located predominantly in small towns and villages in North East England (Table 1); the surrounding areas were ranked from 8.5% to 91.8% on the Index of Multiple Deprivation, a global indicator of socioeconomic status for all areas in England [51], with four of the homes being ranked below the 50th percentile (lower ranks represent greater deprivation). The standard of care provided was rated by the UK Commission for Social Care Inspection as either good (four homes) or excellent (one home).

The homes were self-selected following discussions with senior managers. Participating homes catered for between 25 and 40 clients, the majority of whom were permanent residents. Additional services provided were respite care (five homes), day centers (four homes), and community meals for day centers, lunch clubs, or meals on wheels (four homes). The internal organization of the homes varied: three had a central dining room where cooks served meals to clients and two were unitized, with meals being served by care staff in small dining rooms. One unitized home (home 1) had dedicated staff in each unit.

### Participants

We aimed to recruit a maximum variation sample of staff, that is, staff with different responsibilities and diverse views on existing menus and nutrition guidelines [52]. We included home managers, who had overall responsibility for the food provided; senior staff and head cooks, who were responsible for menu development and food ordering; catering staff, who prepared and served meals; and care and domestic staff, who served food, collected client feedback, and cleared mealtime waste. The use of observation and informal conversations enabled us to engage with a wider range of staff than would have been possible had we relied solely on formal interviews and also facilitated the identification of potential interviewees.

The wider study included interviews with service users and other stakeholders; these are reported elsewhere [35].

### Data collection

Data were collected between April 2008 and June 2010 by two experienced researchers (CB and BH). Interviews were electronically recorded (with consent) and transcribed verbatim. Where participants did not wish to be recorded, the researcher made contemporaneous notes and subsequently wrote a detailed account of the discussion. Some participants were interviewed in pairs or small groups. Topic guides were informed by NPT [53] and were revised to include issues that emerged as important in early interviews. For example, resistance to external guidelines was a strong theme in home 2; we therefore explored staff confidence in government guidelines in subsequent homes. Copies of baseline and modified menus, the nutrition guidelines, and their underlying principles were also used to prompt discussion.

In each home, we observed food preparation and meal times to identify taken-for-granted work practices and routines. Additional observation and informal discussions with staff provided insight into the culture and values of the home. Data on the process of menu development were collected through observation of meetings, training sessions, and informal discussions between the study dietitian and care home staff. Field notes were written as soon as possible following each period of observation and included thoughts and comments about what had occurred and suggestions for further data collection.

### Data analyses

Data analysis took place in two phases to avoid forcing the data into categories predetermined by the theoretical framework [47,48]. An initial thematic analysis conducted by CB and BH was discussed in data analysis workshops with the other authors and underwent a number of iterations, as new issues emerged at different time points and in different care homes. In the second phase of analysis, we mapped emergent data themes to the NPT framework checking for fit. NVivo 8 (QSR International, Cambridge, MA, USA) was used to manage the large dataset. In view of the volume of data collected, the whole of the dataset was not systematically coded; all field notes were coded together with a purposively selected sample of interviews. We coded interviews with key staff (cooks and managers) and staff with strong views on the nutrition guidelines (either positive or negative) at each time point. We then carefully scrutinized the remaining data to identify deviant cases, amend code boundaries if needed and identify any additional themes not captured by the existing coding frame [54].

The trustworthiness, or credibility, of the study was enhanced by the use of different methods and time points and the emphasis on purposive sampling. The two researchers responsible for data collection worked closely together, reflecting on their experiences of data collection, the process of data analysis, and their role in constructing meaning from the data. A detailed codebook was produced to ensure consistency of coding. The involvement of the other coauthors in data workshops provided additional insights from experts in qualitative research and implementation science (CM) and nutrition (PM).

## Findings

The environmental and social context of each home is described in Table 1, which also summarizes the key outcomes and illustrates how the approach to menu modification evolved over time as strategies were developed and implemented. A total of 112 staff took part in interviews; the role of staff interviewed at each time point in each home is shown in Table 2. Observational data and notes of informal discussions resulted in 146 pages of field notes.

**Table 2:** Number of interviews by role, home, and time

**Home**	**Time period**	**Cooks**	**Senior managers**	**Care staff**
Home 1	Baseline	5	4	7
	One-month follow-up	3	4	4
	Five-month follow-up	3	3	2
	*Total for Home 1*	*11*	*11*	*13*
Home 2	Baseline	5	4	11
	One-month follow-up	4	2	0
	Five-month follow-up	NA^a^	NA^a^	NA^a^
	*Total for Home 2*	*9*	*6*	*11*
Home 3	Baseline	4	3	2
	One-month follow-up	1	2	1
	Five-month follow-up	4	1	0
	*Total for Home 3*	*9*	*6*	*3*
Home 4	Baseline	1	3	4
	One-month follow-up	1	1	6
	Five-month follow-up	2	2	2
	*Total for Home 4*	*4*	*6*	*12*
Home 5	Baseline	2	2	2
	One-month follow-up	2	1	2
	Five-month follow-up	NA^b^	NA^b^	NA^b^
	*Total for Home 5*	*4*	*3*	*4*
All homes	Total	37	32	43

^a^As few changes were implemented in home 2, no five-month follow-up interviews were conducted. ^b^The addition of home 5 was due to the low numbers of clients recruited in earlier homes; there was insufficient time or resources to conduct five-month follow-up interviews in this extra home.

### Factors influencing implementation of modified menus

The findings are presented within the NPT framework, together with illustrative quotations. The source of each quotation is indicated by phase (baseline, menu development, implementation, one- or five-month follow-up), type of respondent, and home. Quotations are from interviews, unless otherwise stated.

### Coherence**—**making sense of nutrition guidelines

The nutrition guidelines and modified menus lacked coherence for many staff who:

contested the value of external guidelines,

perceived them as incompatible with existing goals and priorities, and

questioned the benefits of dietary change for older people.

While some staff viewed external guidelines as a resource for improving care, others argued that menus should be locally derived, primarily between cooks and clients. This resistance to external guidelines was particularly marked in home 2, which was situated in a remote close-knit community:

"I think the County Council want to butt out a little bit and I think the government wants to butt out totally because, let’s face it, they don’t know what goes on in a care home, they don’t, they haven’t a clue. (Baseline, cook, Home 2)"

In addition, some staff perceived tensions between the UK policy emphasis on personalization and choice [55] and the nutrition guidelines. Staff wanted to provide a homely environment in which clients were free to choose favorite foods and dishes and perceived the nutrition guidelines as prioritizing the ingestion of nutrients over the emotional, social, and cultural qualities of food and mealtimes. Food and mealtimes were identified as a central focus of daily routines and a key source of well-being for clients:

"In a place like this […] I think food is number one, on the top of the list really, of what they like and they look forward to the most, so I think it’s important that we get it right. (Five-month follow-up, care staff, Home 1)"

The original title of the study (*Healthier Menus in Care Homes*) contributed to this perception, as many staff associated “healthy eating” with dieting, deprivation, and weight loss.

A final reservation voiced repeatedly by staff was that it was “too late” to change the habits of older people and modifying their diet would be of little benefit:

"I know it sounds awful saying this, but if you make the wrong choice at 80-odd, 90-odd year old on what you’re eating, does it matter as much as making the wrong choices when you’re 10, 15, or whatever; there’s a difference isn’t there? I know that sounds ageist but to me there is a difference. (Baseline, senior staff, home 2)"

These concerns over the legitimacy and potential impacts of the nutrition guidelines meant that they did not make sense to significant numbers of staff. This lack of coherence was a significant barrier to implementation, to the extent that the cooks in Home 2 refused to implement the modified menus. The “real” and “ideal” conditions for making sense of the nutrition guidelines and strategies adopted are summarized in Table 3.

**Table 3:** Coherence—real and ideal conditions for making sense of nutrition guidelines

**Real conditions**	**Ideal conditions**	**Strategies to promote coherence**
Value of external guidelines questioned	Recognition that external guidelines may be a useful resource	Shift from *implementing* the nutrition guidelines to *moving towards* the guidelines
Perceived incompatibility with existing goals and priorities	Understanding of ways of improving nutrition while still offering choice and recognizing the emotional and cultural aspects of food and mealtimes	Change study title (from *Healthier Eating in Care Homes* to *Eating for Well*-*being in Care Homes*)
		Keep local and traditional dishes on the menu (adapting recipes rather than menus)
		Focus on occasional treats
Scepticism over the value of changing the diet of care home clients	Recognition of potential benefits to clients	Provide data emphasizing the short-term benefits to clients
		Briefing meetings to introduce the nutrition guidelines to all staff

### Cognitive participation—investing in nutrition guidelines

The uncertainties over the legitimacy and value of the nutrition guidelines clearly impacted the willingness of staff to sign up, or engage with, implementation. Additional barriers to individual and collective investment in the nutrition guidelines were

satisfaction with existing menus,

perceived threats to autonomy and expertise, and

a lack of focus or impetus for implementation.

Reservations about the existing menus were expressed in all homes, and the nutrition guidelines were viewed as a catalyst for change. Typical concerns were that clients were given too many treats and too much to eat, which impacted their mobility, energy levels, and weight:

"So you walk in after breakfast and they’re all sleeping, they’re programmed to come and have their dinner, then they have their heavy dinner, then when you go back in after dinner they’re sound asleep. You can’t do anything with them because they don’t want to. (Baseline, care staff, Home 2)"

Other staff took pride in the existing menus and were reluctant to make changes, particularly where the home enjoyed a good local reputation for the food provided.

A further barrier to investment in nutrition guidelines was the perception that staff skills and expertise were not valued. Some cooks found suggestions to amend tried and tested recipes disempowering and insulting:

"They’re saying, “Well, we’ll come in and make cakes differently.” Well, what was wrong with the way they were made before? Are we not doing our job properly here? It’s quite a difficult one to actually put into words, how you feel […] I just feel as if you’re being undermined somehow. (Baseline, cook, Home 2)"

Some care staff perceived changes as disrupting and devaluing their personal relationships with, and detailed knowledge of, clients. This was a particular issue in home 1, where unit staff “know their clients very, very well—probably better than their own parents” (Baseline, senior staff, Home 1). Observation of mealtimes in this home indicated that staff rarely explicitly asked clients about their preferences, instead automatically adjusting the content and portion size to suit individual clients. Suggestions to explicitly offer clients brown bread before white, water before juice, and polyunsaturated margarine before butter were rejected on the grounds that they would create a less homely, more institutional ambience.

It might have proved possible to create collective investment in the nutrition guidelines despite the diversity of staff views had there been strong management support. In most homes, however, there was little internal focus or impetus relating to the study. The organizational culture of the care homes did not foster widespread debate and discussion; instead, interactions centered on preexisting social networks, which tended to reinforce existing perceptions of the study. The exception was in Home 5, where there was an emphasis on ensuring all staff were on board:

"I think if you don’t train everybody up, then it just gets very confusing and the message gets passed around and it just gets distorted along the way, doesn’t it? By the time it gets down to the domestic, everybody has been put on a diet. (One-month follow-up, senior staff, Home 5)"

The lack of coherence of the nutrition guidelines resulted in staff reluctance to invest in implementation, and this was compounded by the factors described above. Real and ideal conditions for fostering the engagement of staff and strategies to facilitate investment are summarized in Table 4.

**Table 4:** Cognitive participation—real and ideal conditions for investing in nutrition guidelines

**Real conditions**	**Ideal conditions**	**Strategies to promote cognitive participation**
Varied views on existing menus	Scope for improving existing menus widely recognized	Provide feedback on nutritional content of baseline menus
		Highlight role of modified menus in managing diabetes
Perceived threats to autonomy and expertise	Control over pace, extent, and nature of changes to menus/recipes	Delegate responsibility for drafting revised menus/recipes to cooks
		Provide training for all staff
Lack of leadership for implementation	Key individuals take a lead role in creating and sustaining momentum for change	Extend principle of ownership by involving care staff in the process of menu development
	Active support of senior managers with practical issues and in managing any negative feedback on changes	

### Collective action—implementing the nutrition guidelines

The cooks were inevitably largely responsible for the practical work of developing and implementing the modified menus, although care staff also had a role in enacting changes, particularly in unitized homes where they were responsible for serving food. Barriers to practical implementation of the nutrition guidelines included:

limited knowledge of the nutritional content of food,

lack of resources for implementation,

complex and unreliable procurement systems, and

lack of monitoring of implementation.

A consistent barrier in all homes was that staff responsible for developing menus (usually the head cook and a senior manager) lacked detailed knowledge of the nutritional content of foods and the nutritional needs of older people. Although some cooks had an interest in healthy eating on a personal level, nutritional knowledge was variable and was not always considered in the context of work. While all cooks were aware of guidelines on “five a day” [56], potatoes were incorrectly seen to count towards portions of fruit and vegetables by at least one cook. Few cooks or care staff were familiar with the “eatwell plate,” which provides guidance on the relative portions of different food groups required for a healthy diet [57]. The training provided by the study dietitian went some way to improve knowledge and was often valued by the cooks:

"It’s opened my eyes to a lot of things that I knew nothing about really; I’ve found it quite interesting. (Five-month follow-up, cook, Home 3)"

The process of menu development, preparing new dishes and changing the orders created significant extra work. The situation was exacerbated by staff shortages in four homes. As a result, the cooks in all but one of the homes (where staff were more unionized) attended meetings on their days off and did additional work at home (*e*.*g*., reviewing draft menus):

"We weren’t given any extra time for the extra work; we could have done with a bit of help from management and like I say [name of cook] did a lot of work out of hours at home and never got reimbursed or got the lieu days for that. (Five-month follow-up, cook, Home 4)"

The procurement systems used by the County Council meant that cooks were reliant on specific suppliers for fruit and vegetables, meat, baked goods, and general supplies. Since the ordering and delivery dates varied between suppliers, changing the menus was not straightforward. The restrictions on ordering meant staff were reliant on food that was seen as incompatible with the nutrition guidelines:

"They buy in cheap mince but it’s only fat, you’re not getting any more mince, they’re just getting more fat. So at the end of the day, you’re ending up with less mince than you would if you bought a leaner mince. (Baseline, cook, Home 3)"

There were no formal systems for monitoring implementation; following development of the modified menus, staff were individually responsible for putting them into practice. A few members of staff actively resisted implementation, either by refusing to make changes, making changes in ways that were likely to be unacceptable to clients, or sabotaging implementation (*e*.*g*., by “losing” the modified menus). Within all homes, the extent to which different cooks complied with the modified menus varied:

"[Study dietitian] asked whether they were using the polyunsaturated margarine in sandwiches and on toast. One cook said that he had been using it with no problems; the other cook commented that he mixed it half and half with butter for sandwiches, but also said that you “couldn’t put it on toast.” (Menu development, field notes of meeting between cooks and study dietitian, Home 5)"

This lack of consistency often reflected the cooks’ personal preferences and the extent to which they were signed up to the nutrition guidelines, rather than necessarily reflecting client preferences.

Similar variation in support for the modified menus was evident amongst care staff, particularly in relation to serving fruit instead of biscuits with coffee and tea. While some care staff simply left the fruit platter on the trolley, others took an active role:

"I think it’s how they [fruit platters] get presented, but I also think it’s the feedback from the staff when they’re serving them, you know, “How nice does this look? I’m going to have a piece of that pineapple for my tea” […] it’s just the way you promote it. (One-month follow-up, care staff, Home 5)"

Despite their reservations about the value of the nutrition guidelines, the majority of the cooks showed considerable commitment to developing modified menus and made some changes. The real and ideal conditions needed for implementation and strategies to promote the enactment of the nutrition guidelines are summarized in Table 5.

**Table 5:** Collective action—real and ideal conditions for implementing nutrition guidelines

**Real conditions**	**Ideal conditions**	**Strategies to promote collective action**
Inadequate knowledge of nutritional content of foods among cooks and care staff	Consistent understanding of nutritional content of foods, the principles of menu development, and strategies for adapting recipes	Provide detailed training for cooks
		Provide basic training for care staff
		Access to study dietitian to support changes
Additional workload absorbed by existing resources	Employment of supernumerary staff to manage additional workload	Negotiate with County Council for payment for cooks for time spent on menu development
	Dedicated time for existing cooks to work on menu development	
Complex and unreliable procurement systems	Adjust procurement systems to ensure access to required ingredients/foods	Liaise with County Council to revise supply list
	Provide starter pack for homes containing small quantities of new products	Provide cooks with codes of preferred ingredients/foods
Inconsistent systems for monitoring implementation (reflected in variable practice between cooks)	Consistent, agreed-upon approach between cooks	Engage all cooks in training and drafting revised menus/recipes
	Monitoring of implementation	Provide feedback on nutritional content of baseline and modified menus
		See strategies for improving coherence and cognitive participation

### Reflexive monitoring—regaining ownership and embedding changes

To successfully embed nutrition guidelines, staff need to review their experiences of implementation and, if necessary, adapt the modified menus to suit local circumstances. Barriers to reflexive monitoring were

lack of systematic feedback on the impacts on client well-being,

concerns over the reliability of feedback mediated by care staff, and

lack of confidence in modifying menus and recipes.

While the nutrient profile of the modified menus was analyzed for the study, the results were not systematically fed back to participating homes. Staff were therefore largely reliant on their subjective impressions of the impact on clients:

"I wouldn’t say their health has improved any or deteriorated any, I think it’s just nothing has benefited or come out, there’s no outcome yet. (Five-month follow-up, senior staff, Home 1)"

Only two changes were attributed by staff to the modified menus. In home 1, the rapid introduction and high fiber content of the modified menus led to some clients experiencing loose bowels, causing embarrassment, discomfort, and additional work for staff. While loose bowels are associated with health benefits, the introduction of additional fiber needs to be carefully managed to avoid loss of dignity. A reduction in the number of client falls was tentatively linked to the modified menus in one home:

"We analyze accidents and falls in the home every month, and we have noticed over the last few months they’ve steadily gone down and reduced and that could be due to diet and drinking more water and such things. (Five-month follow-up, senior staff, Home 3)"

Staff often interpreted outcomes in line with their preconceptions about the nutrition guidelines. Cooks in unitized homes who relied on care staff for feedback expressed concern over the reliability of staff reports:

"[Cook] added that she thought the main barriers might actually be the care staff “as some of them are overbearing really.” [Second cook] agreed with this statement and added that “some say the clients don’t like something just because they [the carers] don’t like it.” (Baseline field notes, informal discussion with cook, Home 1)"

Initial implementation was followed in all homes by a period where the cooks adjusted the menus or recipes in the light of feedback from clients and/or care staff. This process highlighted the limitations of the training. While some cooks simply reinstated popular dishes from the baseline menus, others tried to follow the principles underlying the modified menus. However, they found it difficult to manage the tension between meeting the nutrition guidelines and client preferences:

"It’s trying to give the clients what they want; you know that’s the hardest thing that I find, for all you can try to say it’s healthy and to look at the nutritional side as you can, they want certain things. (One-month follow-up, cook, Home 4)"

Most of the cooks reported paying more attention to the nutritional content of meals and some had adapted their usual practice as a result:

"I would say I’m probably more aware of nutrition now, I would say because I never used, to be honest with you, really think about it. (Five-month follow-up, cook, Home 4)"

"I think a lot of it is the habits that you get into […] We don’t put butter into the potatoes now, I put a little bit of that margarine stuff and maybe a little drop of milk but before I was just throwing a block [250 g] of butter in. (One-month follow-up, cook, Home 5)"

For some staff, the experience gained from implementing the nutrition guidelines led to new insights and understandings; for others, their views on the value and impact of the modified menus remained unchanged, reflecting the paucity of evidence that clients had benefitted from the modified menus. The real and ideal conditions needed to enable staff to evaluate and adapt the nutrition guidelines to suit local preferences and strategies to facilitate reflexive monitoring are summarized in Table 6.

**Table 6:** Reflexive monitoring—real and ideal conditions for appraising nutrition guidelines

**Real conditions**	**Ideal conditions**	**Strategies to promote reflexive monitoring**
Emphasis on adverse events and lack of systematic feedback on impacts of nutrition guidelines	Access to information on a wide range of outcomes (*e*.*g*., waste, falls)	See strategies for improving coherence and cognitive participation (see Table 3 and 4)
Feedback from clients to cooks mediated by care staff (and potentially contaminated by their own views of the nutrition guidelines and modified menus)	Direct feedback from clients to cooks	Provide “taster” sessions as a way of involving clients and obtaining feedback
		Encourage care staff to separate their own views from those of clients
Lack of information on nutrition profile of modified menus	Comparative information on nutrition profile of baseline and modified menus available	Provide feedback comparing nutrition profile of baseline and modified menus
Cooks lack confidence in adapting menus and recipes (particularly in ways that are acceptable to clients)	Cooks have skills and confidence to update menus and dishes in ways that are consistent with principles underlying the nutrition guidelines *and* acceptable to clients	Provide training in principles underlying the nutrition guidelines
		Provide taster sessions for clients

### Implementation processes and strategies

Our experience of working sequentially in five care homes provided evidence that some aspects of the nutrition guidelines became fully integrated into work practices. Across all homes, the most successful and enduring changes were those that went unnoticed by clients. For example, substituting polyunsaturated for saturated margarine in baking was reported to improve the texture of cakes and have no discernible impact on taste. A gradual reduction in the sugar content of cakes proved acceptable to clients:

"I do think beforehand we were far too heavy-handed with the sugar and everything else, I really do hold my hands up to that because tasting it halfway through, with just adding half the sugar, I think it’s much better. (Five-month follow-up, cook, Home 1)"

The training and briefing sessions for all staff were generally well received and staff seemed better informed about the purpose of the nutrition guidelines in later homes. The shift in emphasis from changing the menus to changing recipes successfully addressed staff concerns over the emotional and cultural aspects of food and was particularly successful in home 5, where clients were largely unaware that the menus had been modified. In general, staff in homes 3 to 5 had more positive attitudes to the nutrition guidelines, with some staff recognizing their wider relevance, suggesting that the strategies to improve coherence and cognitive participation had been successful:

"It’s getting it in your head that it’s actually not a diet, it’s a lifetime commitment to your well-being and I think that’s hit a lot of the staff that wherever you go, it’s not just work-related, this diet, it’s across the board, it’s for your children, it’s for your granny, it’s for you, it’s universal isn’t it? (Six-month follow-up, senior staff, Home 3)"

Devolving responsibility for menu development met with varying success (see also Table 1). In some homes, the majority of the work was carried out by catering staff; in others, staff were content to limit their input to commenting on the draft menus produced by the study dietitian. Initially we focused on engaging the cooks, only including care staff in the process of menu development in the final home. Informal discussion of this strategy with staff in other homes suggests that the careful selection of care staff is key to successful joint working with catering staff.

As an external study team, we were concerned about the lack of resources and management support for implementation but felt relatively powerless to address these issues. While we negotiated reimbursement for staff time with the senior manager at the County Council, the managers of participating care homes proved reluctant to use their budget to pay cooks for their work on menu development. Improving nutrition only appeared to be a priority if it could be achieved within existing resources.

It proved difficult to enhance feedback systems, and there was little evidence of benefits to clients resulting from implementation of the nutrition guidelines. In the absence of other information, cooks valued information we provided on the nutrition content of the baseline and modified menus:

"You’re just working away and you don’t know if you are making a difference or not. But if you have it in black and white […] it makes you proud to think that you’re making a massive difference really to somebody’s life. (Five-month follow-up, cook, Home 3)"

## Discussion

The implementation of menus based on nutrition guidelines in UK care homes proved challenging, although some changes were successfully embedded in routine practice (*e*.*g*., substituting saturated with polyunsaturated margarine in baking). It proved difficult to build collective understanding of and commitment to the study, resulting in inconsistent implementation; similar issues with lack of compliance with nutritional interventions in care homes have previously been reported [46]. The four key constructs of NPT [44] proved useful in understanding the barriers to implementation. Most previous studies using NPT have focused primarily on collective action [45,49]; our work highlights the importance of the remaining constructs, in particular, the critical role of coherence. In home 2, where the cooks refused to implement the modified menus, our experience can be conceptualized as a recursive loop, whereby the failure of the intervention to make sense (coherence) and to engage staff (cognitive participation) resulted in some staff acting out their resistance (collective actions) and bringing about outcomes that fulfilled their expectations, in a self-fulfilling prophecy (reflexive monitoring).

While we used the constructs of NPT to understand the findings, the barriers identified are largely consistent with previous work on guideline implementation. The priority given to personal knowledge over scientific evidence by care staff [58,59] led to some staff contesting the value of the nutrition guidelines. Issues relating to role conflict and perceived incompatibility with other goals [60-63] have undermined the implementation of guidelines on lifestyle management [64,65] and nutrition in other contexts [33,66]. Given this uncertainty over the legitimacy of the nutrition guidelines, the concept of relative advantage was key [61-63]. However, staff who were satisfied with existing menus were more attuned to the potential risks of implementation, particularly given the status of food and mealtimes as the “highlight of the day” [67]. In this context, the lack of observable benefits was a significant barrier. These factors individually and collectively undermined the coherence or sense of the intervention for many staff, leading to a lack of investment in the nutrition guidelines. The situation was exacerbated in most homes by the absence of strong leadership, which is well-established as a facilitator of guideline implementation (*e*.*g*., [33,61,68].

Previous initiatives to improve nutrition have often provided additional staff to deliver aspects of the intervention (*e*.*g*., [15,69]). Although the study dietitian provided training and facilitated the process of menu development, the day-to-day implementation of the modified menus had to be achieved within existing resources. Managers’ commitment to the nutrition guidelines did not extend to using scarce resources to facilitate implementation. Despite the importance of supporting guideline implementation with additional financial and human resources [15,32,58,61,70], we were unable to secure these. The lack of nutritional knowledge and reliance on personal knowledge documented in previous studies [58,71] were also identified in the present study; furthermore, the limited training provided, while valued, was insufficient to enable cooks to modify menus and recipes without the continued support of the study dietitian.

### The value of Normalization Process Theory

The process of using NPT to identify real and ideal conditions for implementation [47] was useful in identifying potential strategies to address the barriers identified. One possible area for further development of NPT would be to link the theoretical constructs of NPT to specific behavior-change techniques; this would increase the practical utility of the theory. NPT highlighted barriers related to the work of implementing the nutrition guidelines; using an alternative theoretical framework, such as the Promoting Action on Research Implementation in Health Services (PARiHS) framework [72], might have directed our attention more to the process of facilitation, in particular, the skills and attributes required for facilitation (including understanding, nurturing staff, and support for learning [72]), but would not necessarily have enabled us to identify so clearly issues relating to the lack of coherence of the nutrition guidelines.

### Implications for wider implementation of the nutrition guidelines

Strategies that may facilitate implementation of nutrition guidelines include:

ensuring that all staff are well briefed on the rationale for, and short- and long-term benefits of, the nutrition guidelines (coherence);

facilitating ownership of the modified menus (to the degree preferred by staff) and focusing on working towards rather than implementing the nutrition guidelines (cognitive participation);

providing ongoing training in the principles underlying the nutrition guidelines, menu analysis, and strategies for adapting recipes (collective action);

agreeing on outcome measures and a process for collecting information to review the impacts of the modified menus (reflexive monitoring).

An implementation team with a broad range of skills is needed to effectively implement these strategies, in addition to adequate resources. While not explored in the present study, policies on procurement of ingredients merit further exploration, since the most successful and enduring changes resulted from simple substitution of ingredients [35].

### Limitations of the study

We studied five care homes in the North East of England. Many of the factors influencing implementation of the nutrition guidelines were identified in all of the homes. The emergence of some new factors in Homes 4 and 5, however, suggests that data saturation may not have been achieved. While the sample of homes was diverse in terms of organization and socioeconomic status, they were public sector homes in one geographical region. Additional factors influencing implementation may emerge in privately run homes and those catering to more diverse client groups.

Facilitation was primarily provided by the study dietitian, who typically worked with individuals or groups at the contemplation or action stage in the cycle-of-change model [73]. In the present study, many staff were not at this stage; a greater emphasis on facilitation activities targeted at planning for change [74] might usefully have addressed staff reservations about the nutrition guidelines.

## Conclusions

The legitimacy and value of nutrition guidelines for older people living in care homes was disputed by significant numbers of staff, resulting in a lack of engagement with and commitment to the study. Practical implementation of the nutrition guidelines was challenging due to the lack of nutritional knowledge of cooks and limited institutional support. The successful implementation of the nutrition guidelines requires that the fundamental issues relating to their perceived value and fit with other priorities and goals be addressed. Specialist support is also needed to equip staff with the technical knowledge and skills required for menu analysis and development and to devise systems to monitor and use information on the impacts of modified menus.

## Competing interests

The authors declare that they have no competing interests.

## Authors' contributions

CB and BH were jointly responsible for data collection and analysis. CB drafted the manuscript. CM participated in the design of the study and contributed to data analysis. PM conceived of the study, participated in its design and coordination and contributed to data analysis. All authors commented on draft manuscripts and approved the final manuscript.
